# Study on the Gut–Brain Mechanism of Escitalopram for Alleviating Symptoms of Disorders of Gut–Brain Interaction in the Elderly—A Cohort Study

**DOI:** 10.3390/jcm15135100

**Published:** 2026-06-30

**Authors:** Qiao Tang, Jing Li

**Affiliations:** Mental Health Center, West China Hospital of Sichuan University, Chengdu 610041, China; m18384592771_1@163.com

**Keywords:** disorders of gut–brain interaction, escitalopram oxalate, depression, anxiety, gastrointestinal symptom, 16S amplicon sequencing

## Abstract

**Objective:** Disorders of gut–brain interaction (DGBIs) are characterized by functional impairments without identifiable organic causes, with their prevalence increasing with age. Emerging evidence suggests that selective serotonin reuptake inhibitors (SSRIs), such as escitalopram oxalate, may influence DGBIs through the brain–gut axis, though the precise mechanisms driving their therapeutic effects remain unclear. This study investigated the impact of escitalopram oxalate on elderly patients with DGBIs in an outpatient department to elucidate these mechanisms. **Methods:** This study was an observational cohort study. We recruited elderly patients diagnosed with DGBIs. Patients receiving standard treatment alone were assigned to the control group, while patients receiving standard treatment plus 10 mg of escitalopram oxalate daily were assigned to the exposure group. Emotional and gastrointestinal symptoms were assessed at baseline and after 12 weeks of treatment using validated symptom scales. Additionally, stool samples were collected at both time points and analyzed via 16S amplicon sequencing to evaluate the changes in gut microbiota. **Results:** A total of 83 elderly patients with DGBIs were included in the study, comprising 40 patients in the control group and 43 in the exposure group. After 12 weeks, the exposure group showed significantly greater reductions in their scores on the Gastrointestinal Symptom Rating Scale (GSRS), Short-Form Leeds Dyspepsia Questionnaire (SF-LDQ), Zung Self-Rating Depression Scale (SDS) and Zung Self-Rating Anxiety Scale (SAS) compared with the control group (e.g., GSRS: 17.00 ± 0.85 vs. 22.58 ± 3.18, *p* < 0.001; *p* < 0.01 for all other scale comparisons), with higher effective and recovery rates. Notably, the exposure group showed significant alterations in the abundance of four genus-level taxa (Blautia, Butyricicoccus, Prevotellaceae UCG-003, and Streptococcus) and two species-level taxa (Eubacterium-hallii-group and Parabacteroides-merdae). **Conclusions:** The escitalopram oxalate treatment was associated with significant improvements in both emotional and gastrointestinal symptoms in elderly patients with DGBIs. These improvements may be linked to alterations in specific gut microbiota taxa, offering a preliminary hypothesis for further investigating the underlying mechanisms of the gut–brain axis.

## 1. Introduction

Disorders of gut–brain Interaction (DGBIs), previously termed functional gastrointestinal disorders (FGIDs) before the release of Rome IV diagnostic criteria in 2016, are chronic conditions characterized by symptoms such as abdominal pain, nausea, vomiting, bloating, diarrhea, constipation, and early satiety. Previous studies have classified all cases meeting the diagnostic criteria for DGBIs and identified five core subtypes: irritable bowel syndrome (IBS), functional dyspepsia, functional constipation, functional diarrhea, and functional abdominal bloating [1]. According to the latest epidemiological reference, the global prevalence of DGBIs exceeds 40%, with rates of 10.7% reported in internet-based surveys and 20.9% in household-based surveys. Moreover, epidemiological studies have shown that the prevalence of DGBIs increases with age, with household-based surveys reporting rates as high as 35.4% among individuals over 65 years old [2]. At present, population aging is an escalating global challenge. In 2019, the number of people aged 65 and older worldwide was approximately 700 million, a figure expected to double by 2050 [3]. Given this demographic shift, addressing DGBIs in the elderly population has become a pressing global health issue, highlighting the urgent need for targeted interventions and improved clinical management strategies.

Although accumulating evidence suggests that the occurrence of DGBIs is correlated with nutritional imbalances, low-grade inflammation, epigenetic and genetic alterations, and dysregulation of gut–brain signaling [4], the exact pathogenesis of DGBIs remains incompletely understood. Due to their complex and multifactorial pathophysiology, DGBIs present with significant treatment challenges. The current therapeutic strategies often focus on symptom management rather than addressing the underlying causes. DGBIs, as noted in some literature, are also termed disorders of gut–brain interaction, and are believed to result from complex interactions between the gastrointestinal tract and the central nervous system [5]. The bidirectional communication between the enteric nervous system (ENS) and the central nervous system (CNS) is modulated by a complex network of neurotransmitters and neuropeptides [6]. Among these, 5-hydroxytryptamine (5-HT), commonly known as serotonin, is recognized as the most pivotal neurotransmitter within the gastrointestinal tract. It plays an essential role in regulating visceral sensitivity, gastrointestinal secretion, and motor function [7]. Recent research has identified 5-HT_3_ and 5-HT_4_ receptors as the primary subtypes mediating serotonergic regulation of gastrointestinal function [8]. This neuromodulatory system underlies the gut–brain axis, with dysregulation of 5-HT signaling increasingly being implicated in the pathophysiology of DGBIs [9].

Preliminary studies in animals have demonstrated that the levels of 5-HT in the brain tissue, gastrointestinal tract, and serum of DGBI model rats are significantly higher than in those of control rats [10]. Correspondingly, human studies have suggested that some patients with DGBIs exhibit a genetic defect in the 5-HT reuptake transporter (serotonin transporter, SERT) that may be responsible for their symptoms [9]. Moreover, DGBIs are frequently accompanied by psychological comorbidities, such as anxiety, depression, and heightened stress sensitivity. These affective disturbances are not merely secondary to the chronic symptoms, but are increasingly being recognized as integral components of the disorder, reflecting the bidirectional communication within the gut–brain axis [11]. Aberrant 5-HT signaling has been observed in DGBIs as well as in mood disorders, supporting the hypothesis that shared serotonergic pathways underlie both gastrointestinal and emotional symptoms. These findings highlight 5-HT as a promising therapeutic target for DGBIs, particularly in patients with co-occurring psychological distress, and provide a rationale for the clinical use of serotonergic agents, including selective serotonin reuptake inhibitors (SSRIs) and 5-HT receptor modulators [12].

Beyond serotonergic signaling, the gut microbiota represents a fundamental, dynamic component of the gut–brain axis, exhibiting remarkable ecological plasticity across the mutualism–pathogenicity spectrum to shape host physiology and disease susceptibility [9]. Gut bacteria possess diverse adaptive mechanisms, including genomic plasticity, horizontal gene transfer, and context-dependent gene regulation. These mechanisms enable bacteria to respond dynamically to environmental pressures, such as inflammation, antibiotic exposure, and dietary changes, shifting their functional roles from beneficial mutualism to opportunistic pathogenicity under specific ecological conditions [13]. In the context of DGBIs, such dysbiosis-related shifts can disrupt intestinal barrier integrity, trigger low-grade systemic inflammation, and alter microbial metabolite production (e.g., short-chain fatty acids, neurotransmitter precursors), which in turn modulate enteric nervous system function and central nervous system signaling via the gut–brain axis [14]. Similar alterations in gut microbiota composition and microbial signaling pathways have also been observed in neurodevelopmental disorders, such as autism spectrum disorder (ASD), pointing to a dysregulation of the gut–brain axis [15]. Dramatically, this microbial plasticity intersects with serotonergic pathways: dysbiotic microbiota can directly influence serotonin synthesis, reuptake, and signaling in the gut, thereby amplifying the effects of serotonergic dysregulation in DGBIs. Importantly, psychotropic medications, such as antidepressants, mood stabilizers, and antipsychotics [16], are associated with gastrointestinal symptoms and can modulate gut–brain axis signaling.

In the elderly population, age-related physiological changes, particularly the decline in gastrointestinal and immune functions, can significantly impair the absorption and metabolism of nutrients and medications [17]. Additionally, the resilience of the gut microbiota to external stressors is reduced, increasing the risk of impairment of the intestinal mucosal barrier function and inflammatory responses [18]. Escitalopram oxalate, the S-enantiomer of the racemic SSRI citalopram [19], has been extensively studied for its antidepressant and anxiolytic properties. It is characterized by favorable pharmacokinetics, including high oral bioavailability; food-independent absorption; minimal drug–drug interactions, particularly with commonly prescribed gastroenterological agents; and a relatively long elimination half-life of approximately 27–33 h, allowing for convenient once-daily dosing with stable plasma concentrations [20]. Clinical pharmacokinetic studies have revealed no clinically meaningful intergroup differences in escitalopram oxalate metabolic profiles across healthy adults, adolescents, the elderly, and subjects with hepatic dysfunction, consistent with the official specifications from the US FDA drug database [21]. Nevertheless, emerging contemporary evidence suggests that escitalopram may achieve elevated plasma concentrations in elderly individuals; hence, initiating treatment at the standard baseline dosage is recommended for geriatric patients. Some studies have reported an increased risk of QT interval prolongation in elderly patients with cardiovascular disease who receive escitalopram at a dose of 20 mg once daily. As a result, clinicians typically limit the dosage to 10 mg once daily in this population [22]. In clinical gastroenterology practice, escitalopram oxalate is increasingly being considered as a therapeutic option for DGBIs, especially when psychological symptoms are present.

Given the excellent clinical applicability of escitalopram oxalate and its positive effects in the elderly shown in previous studies, this research aims to investigate its therapeutic efficacy and underlying mechanisms in the treatment of DGBIs. Specifically, this study is an observational longitudinal investigation aimed at evaluating the association between changes in clinical symptoms and alterations in gut microbiota composition following escitalopram administration, thereby elucidating the gut–brain axis mechanisms in DGBIs.

## 2. Materials and Methods

### 2.1. Study Participants and Consent to Participate

This study is an observational cohort study. Patients with DGBIs admitted to the outpatient department of the Fourth People’s Hospital of Sichuan Province between December 2021 and November 2022 were considered for inclusion in this study. All participants provided written informed consent.

### 2.2. Ethical Support

This study was approved by the West China Hospital of Sichuan University Biomedical Research Ethics Committee (No. 2021-1456).

### 2.3. Inclusion and Exclusion Criteria

#### 2.3.1. Inclusion Criteria

Participants were eligible for inclusion if they were aged over 60 years; had a diagnosis of DGBI according to the Rome IV criteria; had sufficient cognitive ability to comprehend the assessment scales; and had no history of major surgical procedures within the past three years. Clinical evaluations were conducted jointly by at least two psychiatrists and two gastroenterologists.

#### 2.3.2. Exclusion Criteria

Exclusion criteria included: prior use of other neuroregulators; inability or unwillingness to cooperate with assessments or provide informed consent; presence of severe gastrointestinal, renal, cardiac, hepatic, hematological, neurological, or cerebral diseases; major psychiatric disorders; participation in other clinical trials within the past three months; or use of antibiotics within the same timeframe.

### 2.4. Grouping and Treatment Regimens

All enrolled patients received guideline-based general symptomatic treatment according to the 2020 Chinese Consensus on the Management of Disorders of Gut–Brain Interaction, including four categories of medications tailored to individual symptoms: (1) prokinetic agents (mosapride citrate, 5 mg three times daily before meals, Sumitomo Pharma (Suzhou) Co., Ltd. Suzhou, Jiangsu, China); (2) acid-suppressive medications (omeprazole, 20 mg once daily in the morning, AstraZeneca Pharmaceutical Co., Ltd. Wuxi, Jiangsu, China); (3) antidiarrheal agents (loperamide hydrochloride, 2 mg taken as needed for diarrheal symptoms, Xi’an Janssen Pharmaceutical Co., Ltd. Xi’an, Shaanxi, China); and (4) osmotic laxatives (*polyethylene glycol 4000*, 10 g once daily for constipation, Ipsen (Tianjin) Pharmaceutical Co., Ltd. Tianjin, China). Those who received only general symptomatic treatment were classified into the control group, while those treated with escitalopram oxalate(Lundbeck Pharmaceutical (Beijing) Co., Ltd. Beijing, China) plus general symptomatic treatment were classified into the exposure group. As DGBIs are a psychosomatic disorder, SSRIs such as escitalopram are commonly prescribed by gastroenterologists as a routine treatment for DGBIs rather than for independent psychiatric illnesses. Treatment allocation was determined by the treating gastroenterologists based on clinical assessment of DGBI symptoms; no patients had prior psychiatric diagnoses.

### 2.5. Process and Tools Used

#### 2.5.1. General Data

The demographic and sociological data of the patients were collected, including gender, age, ethnicity, marital status, educational level, body mass index (BMI), smoking history, alcohol consumption, and family history of mental illness.

#### 2.5.2. Questionnaire Tools

##### Adult Eating Behavior Questionnaire (AEBQ)

The AEBQ was developed based on the Children’s and Infants’ Eating Behavior Questionnaire (CEBQ/BEBQ) and comprises 35 items. It comprises two primary dimensions: the Food Approach (FAP) dimension and the Food Avoidant (FAV) dimension. The Food Approach dimension consists of four subscales: Hunger (H), Food Responsiveness (FR), Emotional Overeating (EOE), and Enjoyment of Food (EF). The Food Avoidant dimension consists of four subscales: Satiety Responsiveness (SR), Emotional Undereating (EUE), Food Fussiness (FF) and Slow Eating (SE). Each item is rated on a 1–5 scale. Some items are reverse-scored. The subscale scores are computed by summing the relevant item responses, and the total scores for each dimension are derived by aggregating the respective subscale scores. Higher total scores on the Food Approach dimension reflect a stronger motivation or drive to eat, whereas higher total scores on the Food Avoidant dimension indicate a greater tendency to inhibit or restrict eating. Previous research has demonstrated that the AEBQ is a reliable instrument for measuring adult appetite characteristics, with several of its subscale scores showing significant associations with the body mass index (BMI) [23].

##### Mediterranean Dietary Adaptation Screener (MEDAS)

“Mediterranean cuisine” does not simply refer to the dietary habits of the Mediterranean region, but rather to a healthy dietary pattern. This pattern primarily emphasizes the consumption of vegetables, fruits, lean meats such as poultry, and high-quality nuts and fats, while including a relatively small proportion of grains such as rice. Vegetable oil is typically used as the primary cooking fat. The Mediterranean Dietary Adaptation Screener is a 14-item questionnaire consisting of 2 questions regarding general food consumption habits and 12 questions assessing the frequency of consumption. Each question is scored either 0 or 1, resulting in a total score ranging from 0 to 14. A higher score indicates a healthier dietary pattern [24].

##### Mini-Mental State Examination (MMSE)

The Mini-Mental State Examination (MMSE) is a widely used tool that effectively assesses participants’ intellectual status and the extent of cognitive impairment. It comprises seven domains of cognitive testing, enabling a comprehensive evaluation of various cognitive functions, including orientation, attention, memory, and language ability. The scale consists of 30 questions, with one point awarded for each correct answer and zero points given for incorrect or unanswered items. The total score ranges from 0 to 30. Normal cutoff scores are defined as greater than 17 for individuals with no formal education, greater than 20 for those with primary education, and greater than 24 for individuals with high school education or higher [25].

##### Gastrointestinal Symptom Rating Scale (GSRS)

The GSRS is a widely utilized tool for assessing the severity of gastrointestinal symptoms, comprising a total of 16 items. It is organized into five symptom categories: reflux, abdominal pain, indigestion, diarrhea, and constipation. Each symptom is evaluated using a 7-point severity scale, where 1 indicates the absence of symptoms and 7 indicates extremely severe symptoms. The GSRS demonstrates strong reliability and validity and is broadly applicable across the general population [26].

##### Short-Form Leeds Dyspepsia Questionnaire (SF-LDQ)

The Leeds Dyspepsia Questionnaire is used to assess the frequency and severity of indigestion symptoms. Although the scale demonstrates high reliability and validity, it contains an excessive number of items. Therefore, the Short-Form Leeds Dyspepsia Questionnaire, developed by Fraser et al., is considered more practical. This abbreviated version comprises four domains, each assessed along two dimensions—frequency and severity—yielding a total of eight items. Each item is rated on a 0–4 scale, resulting in an overall score range of 0 to 32. Higher scores reflect greater symptom severity and a more substantial impact on daily functioning [27].

##### Zung Self-Rating Depression Scale (SDS)

The SDS, developed by Zung in 1965, is designed to assess depressive symptoms in adults. It effectively reflects the severity of depression and consists of 20 items, each rated on a scale from 1 to 4. The total raw score is obtained by summing the scores of all 20 items, which is then multiplied by 1.25 and rounded to the nearest integer to yield the standard score. A standard score of 50 or higher suggests the presence of depressive symptoms. Previous studies have demonstrated that the SDS exhibits strong reliability and validity [28].

##### Zung Self-Rating Anxiety Scale (SAS)

The SAS, developed by Zung in 1971, is a widely used tool for assessing anxiety symptoms in adults. It consists of 20 items, each rated on a 4-point scale (1–4 points). The total raw score is calculated by summing the scores of all 20 items, which is then multiplied by 1.25 and rounded to the nearest integer to yield the standard score. The standard score classifications are as follows: mild anxiety (50–59), moderate anxiety (60–69), and severe anxiety (70 and above). Numerous domestic and international studies have demonstrated that the SAS possesses good reliability and validity [28].

#### 2.5.3. Collection of Fecal Specimens and Analysis of 16S Amplicons

##### Collection of Fecal Specimens

The fecal samples were sent to a laboratory for 16S amplification analysis within one month after collection. The collection and preservation method is described in Supplementary Material Figure S1.

##### Analysis of 16S Sequencing

16S rRNA gene amplicon sequencing was performed to assess the intervention-induced changes in microbial community composition and relative abundance between the experimental and control groups, both before and after treatment. The ASV (Amplicon Sequence Variant) clustering method was adopted for analysis. Compared with the traditional OTU (Operational Taxonomic Unit) clustering approach, ASV-based analysis offers higher resolution, greater accuracy, and improved reproducibility. This study employed the QIIME2 classification algorithm. This method is more suitable for ASV research than MOTHUR, with higher accuracy and fewer false positives. After obtaining the ASVs, alpha and beta diversity analyses were performed to assess microbial richness, evenness, and community composition differences. To visualize the intergroup variations in fecal microbial communities, a Principal Co-ordinates Analysis (PCoA) and Non-Metric Multidimensional Scaling (NMDS) were applied to project the high-dimensional compositional data into a 2D diagram. Subsequently, quantitative profiling of specific bacterial taxa was conducted to identify shifts in both the taxonomic identity and relative abundance across groups. The detailed content and quality control plan are provided in the Supplementary Materials.

### 2.6. Statistical Analysis

#### 2.6.1. Statistical Analysis of Questionnaire Data

Statistical descriptions and intergroup comparisons of general demographic information (age, gender, educational level, and BMI) were conducted according to the data type and distribution, with the continuous variables described as the mean ± standard deviation. An independent samples *t*-test was used to compare the demographic variables that met the assumptions of normality and homogeneity of variance, whereas non-parametric tests (e.g., Mann–Whitney U test) were applied to the scale data that violated these assumptions—specifically, those exhibiting non-normal distributions or unequal variances across groups. All tests were double-sided, with a significance level set at 0.05.

#### 2.6.2. Analysis of 16S Amplicon Information

The data were merged using FLASH software (v1.2.11); then, quality control and screening were performed using fastp and Vsearch software (v0.23.2), followed by denoising using QIIME2 software (v2022.2). In the final step, dilution curves and species accumulation box plots were drawn. The PCoA and NMDS dimensionality reduction diagrams were drawn using R software (v4.2.1) to determine the species differences among the four groups at various taxonomic levels, such as phylum, class, order, family, genus, and species (Supplementary Material Figure S2).

## 3. Results

**Figure 1:**
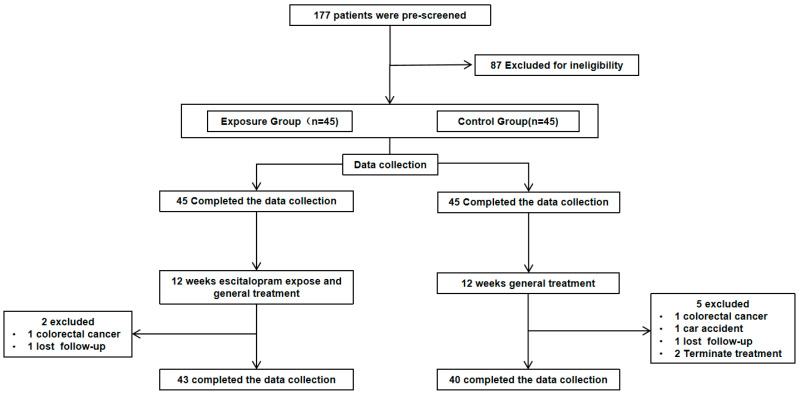
Research flowchart. Note: Arrows indicate the sequential progress of participant screening, grouping, treatment, and follow-up in this clinical trial.

A total of 177 patients were pre-screened, and 87 were excluded for failing to meet the eligibility criteria. The remaining 90 patients were divided into an exposure group (*n* = 45) and a control group (*n* = 45), all of whom completed the baseline data collection. The exposure group received 12 weeks of escitalopram plus routine treatment, with two dropouts (one to colorectal cancer, one loss to follow-up), resulting in 43 patients completing all the data collection. The control group received 12 weeks of routine treatment alone. Five patients were excluded (one to colorectal cancer, one to a car accident, one loss to follow-up, and two to treatment termination), and 40 patients finished the full data collection. The research flowchart is shown in Figure 1.

### 3.1. Comparison of General Demographic Information and Baseline Symptoms

We compared the general demographic characteristics and baseline symptoms of the exposure group and the control group at baseline. There were no statistically significant differences in age, gender, BMI, or educational level between the two groups (all *p* > 0.05). All participants demonstrated normal cognitive function as indicated by their MMSE scores, and no statistically significant differences were found between the exposure and control groups (*p* > 0.05). Furthermore, there were no statistically significant differences between the two groups in their AEBQ or MEDAS scores, nor in the severity of their gastrointestinal and emotional symptoms prior to treatment (all *p* > 0.05, as shown in Table 1).

### 3.2. Comparison of Gastrointestinal Symptoms and Emotional Symptoms After Treatment

All participants exhibited improvements in anxiety, depression and gastrointestinal symptoms following 12 weeks of treatment. The exposure group showed a significantly greater reduction in their clinical symptom scores after 12 weeks of combined treatment with psychotropic medication (*p* < 0.05, as shown in Table 2). Moreover, both the effective rate and clinical recovery rate in the exposure group were significantly higher than those in the control group (*p* < 0.05, as shown in Table 3). (The assessment methods for the clinical effective rate and clinical cure rate of the participants are detailed in the Supplementary Materials).

### 3.3. Comparison of the Differences in the Improvement of Various Gastrointestinal Symptoms Between the Exposed Group and the Control Group

**Figure 2:**
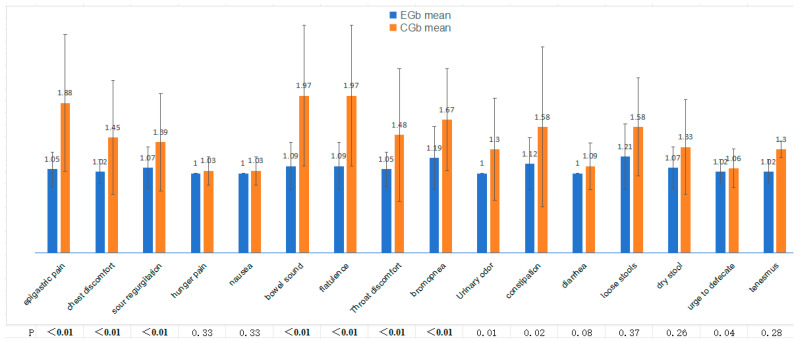
Comparison of individual item mean scores on the Gastrointestinal Symptom Rating Scale between the exposure group and control group after 12-week treatment. Note: Bold values indicate statistically significant differences (*p* < 0.05). *X*-axis serial numbers correspond to GSRS single symptoms: 1. epigastric pain; 2. chest discomfort; 3. sour regurgitation; 4. hunger pains; 5. nausea; 6. bowel sounds; 7. flatulence; 8. throat discomfort; 9. bromopnea; 10. urinary odor; 11. Constipation; 12. Diarrhea; 13. loose stools; 14. dry stool; 15. urge to defecate; 16. tenesmus. EGb: Follow-up at the end of week 12 of exposure group. CGb: Follow-up at the end of week 12 of control group.

The scores for each item on the GSRS were analyzed, with the results presented in Figure 2. After 12 weeks of treatment, both the exposure group and the control group showed improvements in their gastrointestinal and emotional symptoms. A further analysis of individual items revealed that, in the group exposed to escitalopram oxalate, there were significantly greater improvements in symptoms such as upper abdominal pain, chest discomfort, acid reflux, bowel sounds, abdominal distension, throat discomfort, bad breath, abnormal urine odor, constipation, and urgency of defecation (*p* < 0.05).

### 3.4. Changes in Gut Microbiota of Patients with DGBIs

During the research process, a total of 102 fecal specimens that met the research criteria were collected, including 37 from the experimental group and 29 from the control group at enrollment, and 24 from the experimental group (attrition rate 35.1%) and 12 from the control group (attrition rate 58.6%) at the 12th-week follow-up. The fecal specimens were sent for testing within a month. Given the high rate of loss to follow-up for the stool samples, we assessed the potential risk of bias by comparing the baseline characteristics of participants who provided stool samples in the experimental and control groups. No statistically significant differences were observed between the two groups in age, sex, baseline gastrointestinal symptom scores, anxiety and depression scores, or abundance of core gut microbiota (*p* > 0.05). These findings suggested that the stool sample loss was random and unlikely to have introduced meaningful selection bias. To verify the robustness of our core conclusions, we performed a sensitivity analysis. The results showed that although specimen loss to follow-up posed a certain risk of bias, its overall impact was relatively small (Supplementary Materials). Furthermore, we compared the baseline demographic and clinical characteristics of participants who provided stool samples at week 12 and those who did not. No significant differences were observed between the two groups in the key baseline variables (all *p* > 0.05), suggesting that attrition did not introduce systematic bias and the remaining sample was representative of the original cohort (Table 4).

To evaluate the potential confounding effects of omeprazole (PPI) and polyethylene glycol (PEG) on the gut microbiota, the patients who provided fecal samples (*n* = 32) were divided into a PPI/PEG users group (*n* = 20) and a PPI/PEG non-users group (*n* = 12). The abundance of target taxa was compared using an independent samples *t*-test (Table 5). The results showed that no significant differences were found for *Blautia*, *Prevotellaceae_UCG-003*, *Butyricicoccus*, or *Parabacteroides-merdae* (all *p* > 0.05) between the two groups. In contrast, the abundance of the *Eubacterium-hallii group* was significantly higher in the PPI/PEG users group (mean ± SD: 0.0045 ± 0.0044 vs. 0.0020 ± 0.0022; t = 2.144; *p* = 0.040).

The species accumulation boxplot indicated that the sampling depth was sufficient to capture the majority of ASVs. The taxonomic annotation of ASVs revealed distinct compositional differences in the microbial genera between the experimental group and control group both before and after the intervention, which supported the feasibility of subsequent alpha diversity analyses and beta diversity analyses.

#### 3.4.1. The Results of Alpha Diversity Analyses

**Figure 3:**
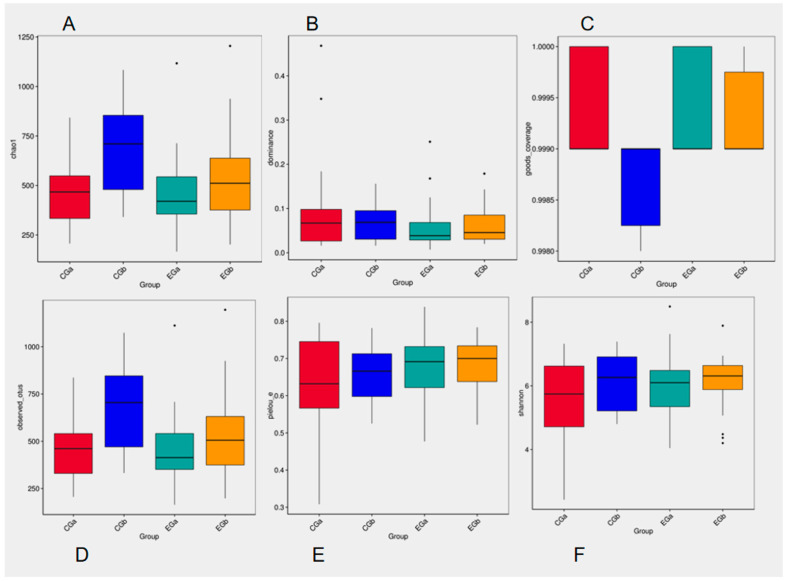
Results of alpha diversity analyses of gut microbial communities in exposure and control groups at baseline and week 12. Note: (**A**): chao1. The size of chao1 index corresponds to the degree of species difference in the community. The larger chao1 is, the more species there are. (**B**): dominance. The probability of two sequences randomly taken from different species. The better the species uniformity of the community, the smaller the index. (**C**): goods_coverage. The higher the sequencing coverage, the larger the index. (**D**): observed_otus. The number of visually observed species. The larger the index, the more species observed. (**E**): pielou_e. Evenness index; the more uniform the species, the larger the index. (**F**): shannon. Total number of categories in the sample and their proportion. The higher the community diversity, the more uniform the species distribution and the larger the Shannon index. EGa: Baseline of exposure group. EGb: Follow-up at the end of week 12 of exposure group. CGa: Baseline of control group. CGb: Follow-up at the end of week 12 of control group.

To explore intergroup differences in species diversity, we compared the number of species between the groups. At baseline, there was no significant difference in species richness between the exposure and control groups (*p* > 0.05). Following treatment, the number of species increased significantly in both groups (Figure 3A,D,F). Additionally, the post-treatment analyses revealed a significant increase in species evenness within the microbial community (Figure 3B,E,F), alongside a significant decrease in sequencing coverage (Figure 3C), with all differences reaching statistical significance (*p* < 0.05).

#### 3.4.2. The Results of Beta Diversity Analyses

**Figure 4:**
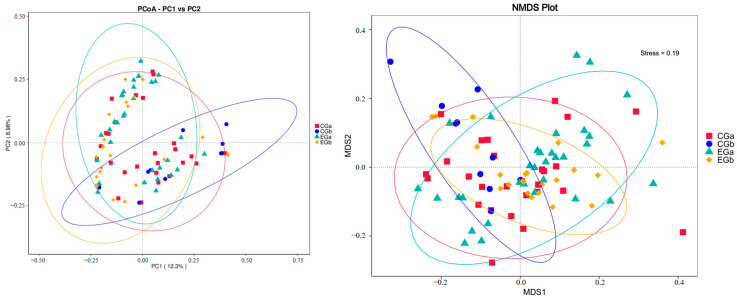
Results of Beta diversity analyses of gut microbial communities in exposure and control groups at baseline and week 12. Note: NMDS: EGa: Baseline of exposure group. EGb: Follow-up at the end of week 12 of exposure group. CGa: Baseline of control group. CGb: Follow-up at the end of week 12 of control group. Each point in the graph represents a sample, and the distance between points indicates the degree of difference. With Stress < 0.2, the results accurately reflect the degree of difference between the samples. The four colored ellipses correspond to the four subgroups defined in the right-side legend.

A Principal Co-ordinates Analysis (PCoA) and Non-Metric Multidimensional Scaling (NMDS) were used to rank the amplified ASVs obtained from all specimens. By plotting, the degree of difference in species composition in the different groups could be intuitively displayed. The results of PCoA and NMDS (Figure 4) both showed significant differences in the microbial community structure of the experimental group and the control group compared to the baseline, with statistical significance (*p* < 0.05).

#### 3.4.3. The Results of Analysis of Bacterial Diversity

**Figure 5:**
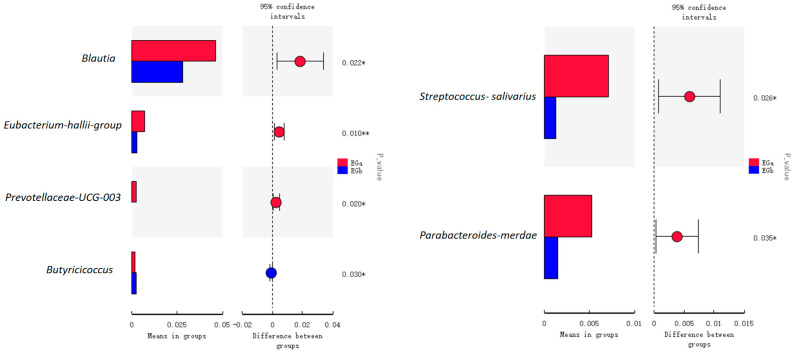
Comparison of relative abundance of differential gut bacterial taxa at baseline and post-exposure in exposure group. Note: *: *p* < 0.05, **: *p* < 0.01. EGa: Baseline of exposure group. EGb: Follow-up at the end of week 12 of exposure group.

The exposure group exhibited notable changes in the composition and abundance of intestinal microbiota before and after exposure, as identified by a *t*-test analysis. Specifically, four genera (*Blautia*, *Eubacterium-hallii-group*, *Prevotellaceae-UCG-003*, and *Butyricicoccus*) and two species (*Streptococcus salivarius* and *Parabacteroides-merdae*) showed significant alterations. The abundance of *Blautia*, *Eubacterium-hallii-group*, *Prevotellaceae-UCG-003*, *Streptococcus salivarius*, and *Parabacteroides-merdae* decreased, whereas the abundance of *Butyricicoccus* increased (Figure 5).

### 3.5. The Results of Correlation Analysis Between Changes in Gut Microbiota and Clinical Symptoms

**Figure 6:**
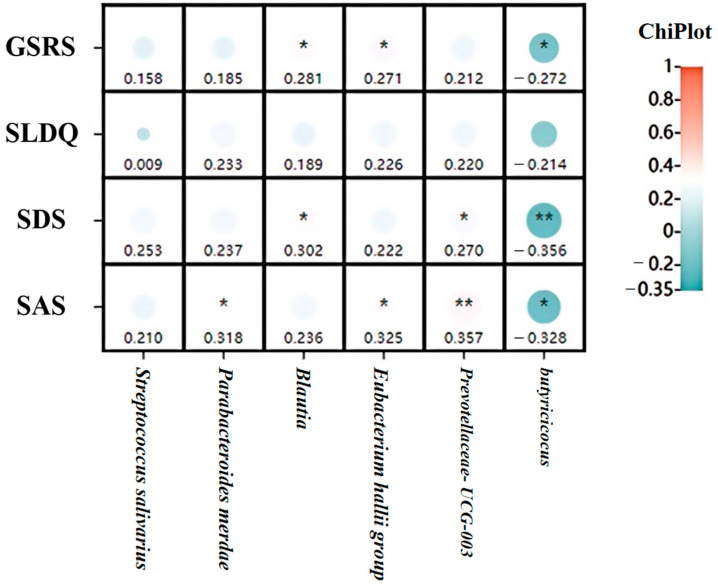
Correlation analysis of microbiota changes and clinical symptoms in the exposure group. Note: Horizontal coordinate: Abundance of 6 bacterial taxa. Ordinate: Total score of 4 scales. The size of the circle and the color depth are proportional to the absolute value of the Pearson coefficient. * *p* < 0.05, ** *p* < 0.01. GSRS: Gastrointestinal Symptom Rating Scale; SF-LDQ: Short-Form Leeds Dyspepsia Questionnaire; SDS: Zung Self-Rating Depression Scale; SAS: Zung Self-Rating Anxiety Scale.

The results regarding the associations between the relative abundances of six bacterial taxa and the symptom scale scores are presented in Figure 6. Specifically, a significant positive correlation was observed between an abundance of *Parabacteroides-merdae* and the SAS scores (*p* < 0.05). Similarly, an abundance of *Blautia* showed a significant positive correlation with both the GSRS scores and SDS scores (both *p* < 0.05). The *Eubacterium-hallii-group* was also positively correlated with the GSRS and SDS scores (*p* < 0.05). In addition, an abundance of Prevotellaceae UCG-003 was positively associated with the SDS (*p* < 0.05) and SAS (*p* < 0.01) scores. Conversely, an abundance of *Butyricicoccus* exhibited a significant negative correlation with the GSRS (*p* < 0.05), SDS (*p* < 0.01), and SAS (*p* < 0.05) scores. Of the six bacterial taxa initially assessed, five showed significant differences in their relative abundances between groups and were included in the final analyses. Detailed quantitative metrics (fold changes, adjusted *p*-values, effect sizes, and 95% confidence intervals) for these five taxa are presented in Table 5. The relative abundances of three bacterial taxa showed significant changes after exposure, even after a Benjamini–Hochberg correction: Blautia (adjusted *p* < 0.01), Eubacterium-hallii-group (adjusted *p* < 0.01), and Butyricicoccus (adjusted *p* < 0.01). Specifically, Blautia and Eubacterium-hallii-group were significantly reduced (fold changes 0.47 and 0.38), while Butyricicoccus was significantly increased (fold change 1.56) (Table 6).

A binary logistic regression analysis revealed that only the relative abundance of *Butyricicoccus* was significantly associated with the GSRS (*p* < 0.05) scores. Detailed results are presented in Table 7.

## 4. Discussion

In this study, we investigated which symptoms of elderly patients with DGBIs are effectively alleviated by Escitalopram oxalate and explored the possible underlying mechanisms. Among them, the exposure group demonstrated significant improvements in symptoms including upper abdominal pain, chest discomfort, acid reflux, bowel sounds, abdominal distension, throat discomfort, and bad breath. After treatment, the types and abundances of bacterial communities in both groups exhibited notable changes. A comparison between the exposure group and the control group revealed statistically significant differences in six bacterial community types. In the exposure group, we found that four types of bacteria were positively correlated with the clinical symptom scores, while *Butyricicoccus* was negatively correlated with the clinical symptom scores. The regression analysis further revealed that, when the demographic information was consistent, an increase in the abundance of *Butyricicoccus* was beneficial for alleviating intestinal symptoms in elderly patients with DGBIs.

In this study, patients with DGBIs exposed to escitalopram oxalate exhibited more significant improvements in their gastrointestinal and emotional symptoms, with a clinical recovery rate of over 90%, while the control group had a clinical recovery rate of less than 40%. The improvement of DGBIs with escitalopram oxalate in the elderly is consistent with prior limited studies [29]. A controlled study showed that citalopram, as a chiral isomer of escitalopram, can alleviate symptoms such as abdominal pain and bloating associated with irritable bowel syndrome [30], which verified the efficacy of escitalopram oxalate on DGBIs from the other side. DGBIs are characterized by a gut–brain interaction dysfunction, accompanied by imbalances in intestinal microbiota, heightened visceral sensitivity, and abnormal gastrointestinal motility. Escitalopram oxalate may be associated with symptom improvement by potentially modulating these pathological profiles. Patients with DGBIs are highly likely to experience long-term serotonin dysregulation; chronic exposure to high concentrations of 5-HT, when combined with 5-HT_3_ receptors on the nociceptive neurons of the colonic and rectal mucosa, can enhance the pain sensation caused by colonic and rectal dilation [31]. Citalopram was shown to be more effective for relieving physical pain than placebo by reducing colonic sensitivity to distension [32]. Moreover, escitalopram oxalate also appears to be beneficial in cases involving constipation. Earlier studies showed that escitalopram can enhance colonic contractility and reduce colonic tension under eating conditions, suggesting its significant therapeutic potential for patients with DGBIs with irritable bowel syndrome. The results of this study also indicate that escitalopram can alleviate the symptom of needing to defecate immediately upon the onset of the urge to do so, a finding consistent with previous research [33]. A detailed analysis of various gastrointestinal symptoms revealed that the improvement in the gastrointestinal clinical symptoms in the exposure group was primarily observed in the upper digestive tract. Previous studies on escitalopram have not reported similar findings. Previous studies have shown that the underlying mechanism may be related to a closer association between upper digestive tract symptoms and visceral hypersensitivity [34]. Under the influence of emotional states such as anxiety and depression, sensory signals from the brain may act on the intestines via the brain–gut axis, thereby increasing visceral hypersensitivity and altering gastric motility and emptying functions [35]. At the same time, these emotions can also trigger abnormal activation of the HPA axis. Previous studies have indicated that dysfunction in the HPA axis may further influence gastric motility and delay gastric emptying [36]. Escitalopram oxalate may have an influence on neural function regulation in both aspects, leading to a more pronounced improvement in the upper digestive tract.

To further investigate the mechanism underlying the changes in upper digestive tract symptoms induced by escitalopram oxalate, we performed 16S rRNA sequencing in the patients with DGBIs before and after treatment. The observed gut microbiota alterations in the exposure group were paralleled by clinical symptom improvement, which may help preliminarily illustrate the potential correlative links between escitalopram treatment, microbial profiles, and gut–brain axis regulation. Previous animal studies have demonstrated that escitalopram can alleviate depressive-like behaviors in rats by regulating gut microbiota and sphingolipid metabolism. This effect is thought to occur through the restoration of intestinal permeability and brain tissue integrity, thereby preventing a reduction in intestinal tight junction proteins, such as claudin-1 and ZO-1, and ultimately exerting a protective role on intestinal barrier function [37]. However, these prior studies did not specify the particular types of microbial changes involved. Our study addresses this gap by providing clinical evidence to complement those findings. In this study, four genera of bacteria, *Blautia*, *Eubacterium-hallii-group*, Prevotellaceae UCG-003, and *Butyricicoccus*, and one species, *Parabacteroides-merdae*, showed significant changes in abundance after exposure to escitalopram oxalate. Previous studies have confirmed that omeprazole (PPI) and polyethylene glycol (PEG) exert prominent and well-documented influences on gut microbiota [38]. In contrast, the available evidence regarding their impacts on gut microbiota remains insufficient. For this reason, we divided the participants into a PPI/PEG users group and PPI/PEG non-users group. In the present analysis, only the Eubacterium-hallii-group exhibited a significant difference between the two groups, while the other four target taxa showed no obvious changes. These results suggest that PPI and PEG have limited impacts on most of the detected bacterial taxa in our cohort. Nevertheless, given the relatively small sample size of participants who provided fecal samples, the above findings should be interpreted cautiously.

*Blautia* is a recently identified core intestinal bacterium that decreases with age, but increases in patients with irritable bowel syndrome. This is consistent with the results of this study [39]. We observed that the clinical symptom scores of patients with DGBIs decreased as the content of *Blautia* decreased, and this reduction was also significantly correlated with increased scores on the SAS and SDS emotional scales. *Blautia* is known to be a major producer of short-chain fatty acids (SCFAs), which play key roles in regulating intestinal barrier function, reducing systemic inflammation, and modulating the gut–brain axis. A decline in *Blautia* abundance may therefore weaken these protective effects, potentially contributing to increased visceral hypersensitivity and mood disturbances, such as anxiety and depression, as reflected in the elevated SAS and SDS scores [40]. It is important to emphasize that these associations were identified through correlation analyses and did not reach statistical significance in our regression models. Therefore, these findings should be interpreted as potential biological clues rather than definitive causal evidence. We speculate that a decline in *Blautia* might weaken SCFA-mediated protective effects, potentially contributing to visceral hypersensitivity and mood disturbances, but this mechanism requires further validation. The research on *Eubacterium-hallii-group* covers many different subjects, such as thyroiditis [41] and diabetes [42]. An analysis of intestinal microbiota of medical workers fighting against the COVID-19 epidemic showed that under a long-term high-pressure stress environment, the *Eubacterium-hallii-group* is closely related to recurrent attacks of post-traumatic stress disorder [43]. The correlation analysis indicated that the level of bacteria in this genus is positively related to stress symptoms. The inconsistency with the results of this study suggests that the *Eubacterium-hallii-group* may have different responses to stress-related reactions and anxiety–depression-related reactions. Thus, it may serve as a biomarker for distinguishing stress reactions from anxiety and depression reactions. Similarly, reductions in *Prevotellaceae-UCG-003* were correlated with higher SAS and SDS scores. Although primarily studied in ruminants, emerging evidence links this taxon to human gastrointestinal and mental health via the degradation of complex carbohydrates and production of beneficial metabolites [44]; its depletion may theoretically disrupt microbial homeostasis and exacerbate inflammatory pathways, though its specific therapeutic role remains unclear based on our current data. Previous studies of *Parabacteroides-merdae* have suggested possible beneficial effects on cardiovascular diseases [45]. But this study suggests that it plays an unfavorable role in DGBIs. Therefore, more detailed research is needed to determine whether it can be used as a probiotic.

The correlation analyses revealed that the abundance of *Butyricicoccus* was positively associated with improvements in gastrointestinal and emotional symptoms, consistent with previous findings on this genus. These results suggest that the therapeutic effects of escitalopram oxalate exposure may partially be mediated by an increase in *Butyricicoccus* levels. *Butyricicoccus* are a common genus of gastrointestinal functional protective bacteria, first identified and reported in 1933, and have been used as live bacterial preparations to regulate intestinal function for over 50 years. *Butyricicoccus* can ferment in the human gastrointestinal tract to produce butyrate salts [46], which are beneficial to the host’s intestinal health. They not only help maintain the homeostasis of the intestinal microbiota, but also compete with and antagonize pathogenic bacteria, reduce the production of Clostridium difficile toxin proteins, inhibit cancer cell proliferation, and induce apoptosis [47]. In addition, they increase intestinal mucin and antimicrobial peptides levels, enhance the expression of tight junction proteins, reduce intestinal permeability, inhibit microbial growth, and prevent translocation of microbial products into the bloodstream [48]. Preclinical animal research demonstrated that supplementation with butyrate-producing *Butyricicoccus* strains effectively repaired gut microbial dysbiosis and relieved DSS-triggered colitis, whose abundance is closely correlated with inflammatory severity in inflammatory bowel disease [49]. Furthermore, a dietary study showed that during the long-term use of a Mediterranean dietary regimen, the content of *Butyricicoccus* will partially increase [50]. These accumulating lines of evidence support the findings of our study. Higher levels of *Butyricicoccus* boost the production of protective butyrate salts in the gut, thereby promoting intestinal homeostasis and alleviating gastrointestinal symptoms. The findings of this study support a beneficial role of *Butyricicoccus* in alleviating both gastrointestinal and emotional symptoms. Although the regression analysis did not yield statistically significant associations between *Butyricicoccus* abundance and emotional symptom improvement, notable positive trends were observed. Importantly, the participants in the experimental group exhibited significant improvements in their emotional symptoms, suggesting a potential indirect neuromodulatory effect. It is speculated that escitalopram may enhance the production of butyrate by increasing the abundance of *Butyricicoccus*, thereby alleviating low-grade intestinal inflammation, enhancing gut barrier integrity, and reducing visceral hypersensitivity, thus alleviating DGBIs. Collectively, these results position *Butyricicoccus* as a promising gut microbial biomarker, offering a valuable direction for future mechanistic studies.

However, the observed association between gut microbiota composition and clinical symptoms in this study may have been confounded by various unmeasured or residual factors and cannot be directly ascribed to the treatment itself. Although there were no significant differences in the baseline demographic characteristics, such as dietary habits, smoking, and alcohol consumption, among the subjects, notable heterogeneity existed in symptom severity and concomitant medication use among the participants. Moreover, this study was conducted at a single hospital center in China. The gut microbiome profiles and dietary patterns of the elderly patients with DGBIs included in this cohort may be strongly influenced by regional dietary habits and local microbial exposure. The findings may not be directly generalizable to other populations, particularly those with different cultural backgrounds, dietary patterns, or healthcare systems. We also acknowledge the bidirectional nature of the gut–brain axis in interpreting our findings. While we observed improvements in gastrointestinal symptoms and parallel changes in gut microbiota following escitalopram treatment, we recognize that reductions in anxiety and depression may themselves contribute to these outcomes. Emotional improvements can reduce visceral hypersensitivity and modulate gastrointestinal motility and secretion, which in turn could influence the composition of the gut microbiota. Thus, the observed changes in microbiota may be secondary to improved mood and gastrointestinal function, rather than a direct effect of escitalopram on the microbiota. Given the observational design of our study, we cannot definitively disentangle these pathways. To sum up, this study offers only a preliminary hypothesis regarding potential gut–brain axis mechanisms. To establish causality and mitigate any confounding bias, more rigorous multi-center randomized controlled trials should be conducted.

## 5. Limitations

There are several limitations of this study.

(1) All the participants were recruited from the same hospital; thus, the sample may not be representative of the broader population. Validation in larger, more diverse cohorts is needed.
(2) With only a single follow-up, it is difficult to capture the course and trajectory of changes over time. Moreover, outpatients often struggle to consistently monitor and report their dietary habits during the study period, making it challenging to adequately control for potential confounding variables.
(3) The clinical manifestations of patients with DGBIs are highly heterogeneous and no structured psychiatric interview was conducted. The study population exhibited a wide spectrum of gastrointestinal symptoms, and symptomatic treatment varied across four distinct drug regimens, introducing potential confounding and bias into the analysis.
(4) Participants were not stratified by specific DGBI subtypes, precluding subgroup analyses and thereby limiting our ability to assess the subtype-specific effects of escitalopram.
(5) As an observational study, this research has not yet clarified the precise mechanism underlying escitalopram’s therapeutic effects: it remains uncertain whether symptom improvement stems primarily from alleviation of mood disturbances or exerts a direct effect on the gut microbiota that subsequently drives symptom relief. Causal relationships cannot be established, and our findings should be interpreted cautiously as preliminary associative observations only. Further rigorous, mechanistic investigation is required.
(6) Although we have made every effort to control all confounding variables, due to the inherent limitations of observational studies, we still cannot completely eliminate interference from all unknown or unmeasured confounding factors.
(7) At the 12-week follow-up, the attrition rate for fecal samples was 35.1% in the exposure group and 58.6% in the control group, representing a substantial loss of biological data, particularly in the control group. This differential dropout may have introduced selection bias and reduced the statistical power of the beta diversity analyses. To address this potential bias, we compared the baseline demographic and clinical characteristics of participants who provided follow-up stool samples and those who did not, and found no significant differences between the two groups. This suggests that the remaining samples were still broadly representative of the original cohort, though the microbiological findings should still be interpreted with caution.

## 6. Conclusions

This study investigated the effects of escitalopram oxalate on the emotional and gastrointestinal symptoms of elderly patients with DGBIs and its underlying mechanisms. Our findings indicate that escitalopram oxalate not only significantly alleviates emotional symptoms but also effectively improves gastrointestinal dysfunction, with the most pronounced improvements observed in upper gastrointestinal symptoms. Analysis of gut microbiota revealed that treatment was associated with reduced abundances of *Blautia*, *Eubacterium-hallii-group*, Prevotellaceae UCG-003, *Streptococcus salivarius*, and *Parabacteroides-merdae*, alongside an increased abundance of *Butyricicoccus*. The multivariable regression analysis identified Butyricicoccus as the only bacterial taxon independently associated with symptom improvement. Given that butyrate supports gut barrier function and reduces inflammation, we hypothesize that Butyricicoccus may contribute to symptom improvement via butyrate production; however, this mechanistic explanation remains a plausible inference based on current evidence and does not establish a causal relationship. These microbial alterations were closely linked to symptom improvement. Given their strong association with the clinical manifestations of Disorders of Gut–Brain Interaction (DGBIs), they may constitute a key component of the gut–brain axis mechanisms underlying symptom development, providing a hypothesis for further exploration. *Butyricicoccus* is a potential target for future interventions, such as fecal microbiota transplantation. However, studies are needed to elucidate the causal relationships and clinical applicability of microbiota modulation in DGBI management.

## Figures and Tables

**Table 1:** Baseline demographic, psychological and gastrointestinal symptom indicators of elderly patients with DGBIs in the exposure group and control group before treatment.

Variables	Exposure Group (*n* = 43)	Control Group (*n* = 40)	t/χ^2^	*p*-Value
Age, years	68.98 ± 8.29	70.63 ± 8.39	−0.900	0.371
Gender			0.242	0.623
Male	16 (37.21%)	17 (42.50%)		
Female	27 (62.79%)	23 (57.50%)		
BMI	21.35 ± 2.80	22.42 ± 3.75	−1.472	0.145
Years of education	10.56 ± 4.01	11.95 ± 4.18	−1.547	0.126
AEBQ (FAP)	48.12 ± 3.44	48.78 ± 3.25	−0.897	0.373
AEBQ (FAV)	58.05 ± 3.74	58.30 ± 3.61	−0.314	0.754
MEDAS score	6.60 ± 2.07	6.73 ± 1.77	−0.284	0.777
MMSE score	27.93 ± 3.61	27.88 ± 2.79	1.969	0.938
GSRS score	36.09 ± 6.98	34.25 ± 5.93	1.292	0.200
SF-LDQ score	11.44 ± 4.37	10.40 ± 4.02	2.211	0.261
SDS score	54.47 ± 7.97	51.95 ± 7.36	1.491	0.140
SAS score	45.60 ± 5.40	43.92 ± 5.85	1.361	0.177

Note: Continuous data are presented as the mean ± standard deviation, and categorical data are presented as the count (percentage). The *p*-value for the gender group is calculated using a chi-square test; the remaining groups are subject to an independent sample *t*-test to calculate the *p*-value. BMI: body mass index; AEBQ: Adult Eating Behavior Questionnaire; MEDAS: Mediterranean Dietary Adaptation Screener; MMSE: Mini-Mental State Examination; GSRS: Gastrointestinal Symptom Rating Scale; SF-LDQ: Short-Form Leeds Dyspepsia Questionnaire; SDS: Zung Self-Rating Depression Scale; SAS: Zung Self-Rating Anxiety Scale.

**Table 2:** Comparison of gastrointestinal and emotional scale scores between the exposure group and control group after 12-week treatment.

Variables	EGb (*n* = 43)	CGb (*n* = 40)	Z	*p*-Value
GSRS score	17.00 ± 0.85	22.58 ± 3.18	−7.811	**<0.001**
SF-LDQ score	0.23 ± 0.43	3.18 ± 2.24	−6.991	**<0.001**
SDS score	28.54 ± 2.59	37.90 ± 7.06	−6.405	**<0.001**
SAS score	26.57 ± 1.67	33.90 ± 4.44	−7.350	**<0.001**

Note: EGb: Follow-up at the end of week 12 of exposure group. CGb: Follow-up at the end of week 12 of control group. The score for each scale is presented as the mean total score ± standard deviation. Bold values indicate statistically significant differences (*p* < 0.05).

**Table 3:** Comparison of clinical effective rate and clinical recovery rate of gastrointestinal and emotional symptoms between the exposure group and control group after 12-week treatment.

Variables	Group	Case	1	2	3	4	Effective Rate/%	*p*-Value	Clinical Recovery Rate/%	*p*-Value
GSRS score	EGb	43	43	0	0	0	100%	**0.032**	100.00%	**<0.01**
	CGb	40	5	27	7	1	97.5%		12.50%	
SF-LDQ score	EGb	43	43	0	0	0	100%	**0.032**	100.00%	**<0.01**
	CGb	40	13	21	5	1	97.5%		32.50%	
SDS score	EGb	43	41	2	0	0	100%	**<0.01**	95.30%	**<0.01**
	CGb	40	2	12	16	10	75%		5.00%	
SAS score	EGb	43	39	4	0	0	100%	**<0.01**	90.70%	**<0.01**
	CGb	40	1	16	15	8	80%		2.50%	

Note: 1, clinical recovery; 2, significant effective; 3, effective; 4, ineffective. EGb: Follow-up at the end of week 12 of exposure group. CGb: Follow-up at the end of week 12 of control group. GSRS: Gastrointestinal Symptom Rating Scale; SF-LDQ: Short-Form Leeds Dyspepsia Questionnaire; SDS: Zung Self-Rating Depression Scale; SAS: Zung Self-Rating Anxiety Scale. Bold values indicate statistically significant differences (*p* < 0.05).

**Table 4:** Baseline demographic, gastrointestinal and emotional characteristic comparisons of patients with fecal samples and patients without fecal samples.

Variables	Fecal Sample Group (*n* = 33)	Non-Fecal Sample Group (*n* = 27)	t/χ^2^	*p*-Value
Age, years	68.67 ± 8.20	69.48 ± 7.62	−0.398	0.692
Gender			−0.479	0.634
Male	13 (39.39%)	9 (33.33%)		
Female	20 (61.61%)	18 (66.67%)		
BMI	21.76 ± 3.28	21.99 ± 3.07	−0.280	0.781
Years of education	11.82 ± 4.13	10.59 ± 3.84	1.190	0.239
MEDAS score	6.67 ± 2.27	6.74 ± 1.51	−0.151	0.881
MMSE score	27.79 ± 4.08	28.07 ± 2.73	−0.324	0.747
GSRS score	35.12 ± 6.38	36.78 ± 5.69	−1.063	0.292
SF-LDQ score	10.12 ± 3.48	11.00 ± 3.62	−0.952	0.345
SDS score	51.91 ± 8.78	49.70 ± 9.73	0.913	0.366
SAS score	44.33 ± 5.77	42.44 ± 4.64	1.405	0.165

Note: BMI: body mass index; MEDAS: Mediterranean Dietary Adaptation Screener; MMSE: Mini-Mental State Examination; GSRS: Gastrointestinal Symptom Rating Scale; SF-LDQ: Short-Form Leeds Dyspepsia Questionnaire; SDS: Zung Self-Rating Depression Scale; SAS: Zung Self-Rating Anxiety Scale.

**Table 5:** Comparison of gut microbiota abundance between patients with and without proton pump inhibitors or polyethylene glycol use.

Taxon	PPI/PEG Users Group (*n* = 20)	PPI/PEG Non-Users Group (*n* = 12)	t	*p*
*Parabacteroides-merdae*	0.0018 ± 0.0021	0.0021 ± 0.0038	−0.186	0.855
*Blautia*	0.0340 ± 0.0243	0.0208 ± 0.0168	1.654	0.109
*Eubacterium-hallii-group*	0.0045 ± 0.0044	0.0020 ± 0.0022	2.144	**0.040**
*Prevotellaceae-UCG-003*	0.0001 ± 0.0006	0.0011 ± 0.0039	−0.984	0.346
*Butyricicoccus*	0.0034 ± 0.0023	0.0024 ± 0.0019	1.319	0.197

Note: PPIs: proton pump inhibitors; PEG: polyethylene glycol. Bold values indicate statistically significant differences (*p* < 0.05).

**Table 6:** Quantitative metrics of bacterial taxa with significant changes in relative abundance before and after exposure in exposure group.

Taxon	Mean Relative Abundance (EGa)	Mean Relative Abundance (EGb)	Fold Change	Adjusted *p*	Cohen’s d (95% CI) for Mean Difference
*Parabacteroides-merdae*	0.0053	0.0015	0.28	0.437	0.6 (0.0028, 0.0073)
*Blautia*	0.0607	0.0282	0.47	**<0.01**	1.03 (0.0125, 0.0525)
*Eubacterium-hallii-group*	0.0080	0.0030	0.38	**<0.01**	0.99 (0.0018, 0.0088)
*Prevotellaceae-UCG-003*	0.0032	0.0006	0.19	0.620	0.52 (−0.0014, 0.0053)
*Butyricicoccus*	0.0016	0.0025	1.56	**<0.01**	−1.05 (−0.0023, −0.0007)

Note: Bold values indicate statistically significant differences (*p* < 0.05).

**Table 7:** Logistic regression analysis linking the relative abundance of key intestinal bacterial taxa to the GSRS total score and the SDS total score of the exposure group after 12-week treatment.

Taxon	GSRS			SDS		
	β	OR (95%CI)	Adjusted *p*	β	OR (95%CI)	Adjusted *p*
*Parabacteroides-merdae*	−0.019	0.982 (0.813–1.186)	0.847	−0.041	0.960 (0.808–1.139)	0.799
*Blautia*	0.301	1.351 (0.497–3.671)	0.694	0.288	1.333 (0.529–3.362)	0.799
*Eubacterium-hallii-group*	0.632	1.882 (0.759–4.662)	0.430	0.341	1.407 (0.687–2.881)	0.585
*Prevotellaceae-UCG-003*	1.916	6.791 (0.001–8.315)	0.998	0.142	1.152 (0.979–1.356)	0.220
*Butyricicoccus*	−1.736	0.176 (0.051–0.605)	**0.030**	−1.025	0.359 (0.146–0.883)	0.130

Note: GSRS: Gastrointestinal Symptom Rating Scale; SDS: Zung Self-Rating Depression Scale. Bold values indicate statistically significant differences (*p* < 0.05).

## Data Availability

The data presented in this study are available on request from the corresponding author. Due to ethical restrictions and patient confidentiality agreements approved by the Institutional Review Board, de-identified data will be made available upon reasonable request, pending approval from the ethics committee.

## Supplementary Materials

The following supporting information can be downloaded at: https://www.mdpi.com/article/10.3390/jcm15135100/s1.

## Abbreviation

DGBIs	Disorders of Gut–Brain Interaction
IBS	Irritable Bowel Syndrome
ENS	Enteric Nervous System
CNS	Central Nervous System
AEBQ	Adult Eating Behavior Questionnaire
MEDAS	Mediterranean Dietary Adaptation Screener
MMSE	Mini-Mental State Examination
GSRS	Gastrointestinal Symptom Rating Scale
SF-LDQ	Short-Form Leeds Dyspepsia Questionnaire
SDS	Zung Self-Rating Depression Scale
SAS	Zung Self-Rating Anxiety Scale
ASVs	Amplicon Sequence Variants
PcoA	Principal Co-Ordinates Analysis
NMDS	Non-Metric Multidimensional Scaling
EGa	Baseline of Exposure Group
EGb	Follow-Up at the End of Week 12 of Exposure Group
CGa	Baseline of Control Group
CGb	Follow-Up at the End of Week 12 of Control Group
