# Supplementary material for: Study on the Gut–Brain Mechanism of Escitalopram for Alleviating Symptoms of Disorders of Gut–Brain Interaction in the Elderly—A Cohort Study
Source: J Clin Med. 2026 Jun 30;15(13):5100. doi: 10.3390/jcm15135100 (PMC13362538; doi:10.3390/jcm15135100)
Supplement: Supplementary file 1 [file jcm-15-05100-s001.zip › jcm-4372842-supplementary.pdf]

Study on the Gut–Brain Mechanism of Escitalopram for Alleviating Symptoms of Disorders of Gut–Brain Interaction in the Elderly—A Cohort Study

Content

Methods.......... 2

1.Clinical symptom assessment method.......... 2

2.Sample size estimation.......... 2

3.Fecal specimens.......... 2

4.16S sequencing.......... 3

5.Analysis of 16S amplicon information.......... 4

Results.......... 6

1.Analysis of amplicon sequence variants.......... 6

2.The differences in ASVs between groups.......... 8

3.Differences in dominant species before and after treatment in the expossure group.......... 9

4.Comparison of demographic data of the subjects who provided stool samples.......... 10

5.Analysis results of demographic factors, mental status, and dietary patterns influencing clinical symptoms.......... 11

Methods

1.Clinical symptom assessment method

Compare the changes in clinical questionnaire scores before and after the intervention between the experimental and control groups to assess improvements in participants' clinical symptoms. Treatment response was categorized based on the percentage reduction in symptom scores as follows:

1.Clinical recovery ≥ 80% reduction;

2.Significant effective: 50% ≤ reduction < 80%;

3.effective: 30% ≤ reduction < 50%;

4.ineffective: < 30% reduction.

2.Sample size estimation

According to previous research results, the allowable error in the score of the Gastrointestinal Symptom Rating Scale (GSRS) in this study is 5 (SD is 10). In order to obtain such differences, based on a bilateral significance level of 0.05 and an efficiency of 80%, we need to include at least 32 subjects in the control group and at least 64 subjects in the experimental group respectively. Considering the dropout rate of about 20% and some results not meeting the requirements of randomized controlled trials, it was planned to include 45 people in each of the two groups for a total of 90 subjects in this study.

3.Fecal specimens

The collection of fecal specimens directly affects the results of 16S amplicon analysis, so the quality control requirements for specimen collection are high. Before collection, the research group doctor provides guidance on the collection method for the subjects and requires them to strictly collect according to the requirements. Fecal specimens that do not meet the quality control standards will be discarded. Note the following: For fresh fecal specimens, use a sampler to take extra samples from the surface, depth, and fecal end, measuring about half the size of a finger joint. Urine, disinfectant, or sewage should not be mixed. After collecting the specimens, they should be completely immersed in the liquid inside the tube. The specimen should be stored at room temperature and sent for 16S amplification analysis within one month.

4.16S sequencing

Species abundance: The species abundance of a sample includes absolute abundance and relative abundance. After the analysis is completed, you can directly use the species abundance table to read and view the species composition and distribution in each sample, or observe the composition of the samples by grouping categories, so as to select the species needed for the next analysis.

Sample complexity analysis (Alpha analysis): The richness and uniformity of species in the sample can be reflected by the alpha diversity index, and the value of the diversity index can reflect the complexity of the microbial community contained in the sample; In addition, the significant difference test results of the alpha diversity index between groups can identify the groups with the most significant changes in species diversity.

Diversity Comparative Analysis (Beta Analysis): Beta diversity can reflect the differences in microbial community structure between samples, further determining whether these differences are consistent with biological grouping, and providing a reasonable explanation for these differences in combination with biological issues.

5.Analysis of 16S amplicon information

In this study, the microbiome methods was designed to compare microbial community composition between the experimental and control groups by using 16S rRNA gene amplicon sequencing. We adopted the ASV (Amplicon Sequence Variant) clustering approach coupled with the QIIME2 classification algorithm, which offers superior resolution compared to traditional OTU (Operational Taxonomic Unit) clustering and Mothur classification algorithms. This strategy has higher accuracy and is less likely to produce false positive results, which particularly advantageous for longitudinal studies with limited sample sizes. Primers are designed targeting conserved bacterial genomic regions to enable specific amplification, followed by paired-end 250 bp sequencing on the upgraded Illumina NovaSeq platform. Raw reads underwent rigorous bioinformatic processing including paired-end read merging, quality filtering, denoising, and chimera removal to generate high-resolution ASVs. During this process, multiple statistical approaches were applied including adjusted t-tests (e.g., Benjamini-Hochberg false discovery rate correction) and LEfSe (Linear Discriminant Analysis Effect Size) to improve the biological interpretability and relevance of the identified ASVs. These methods are consistent with the content of the literature you provided. After that, alpha diversity metrics (e.g., Shannon, Simpson, Chao1) were used to assess within-sample microbial richness and evenness and Beta diversity assessments (e.g., PCoA, NMDS) were used to evaluate between-sample compositional differences both experimental and control group.

Sequencing data processing: Cut and splice the PCR amplified sequences to obtain raw data (Raw Tags), and select FLASH(Fast Length Adjustment of Short reads) software for splicing. FLASH is the core tool for splicing of paired-end Reads in 16S amplicon analysis, used to merge

Illumina PE data into complete amplified fragments, and is a commonly used option for the pre-quality control step of QIMME2. It can improve read quality and facilitating downstream analyses such as amplicon sequence variant (ASV) clustering.Quality control and removal of chimeras are performed on the obtained raw data to obtain effective tags. The specific software choices are fastp and Vsearch.

By comparing the similarity of each pair of sequences, concatenating and filtering the sequences, and then performing noise reduction on the similar sequences, only the sequences that are 100% identical are retained for data clustering to improve the accuracy of sequence alignment. Each sequence cluster obtained was called Amplicon Sequence Variants (ASVs), or feature sequences. ASVs refer to fragments with the same base sequence arrangement. Performing species annotation on these ASVs can significantly reduce the number of operations compared to testing the species of each individual sample one by one. The ASVs of different individuals can reflect the differences in the types and abundances of the intestinal microbiota of each individual.

ASVs noise reduction and species annotation: ASVs and their feature tables require filtering out sequences with abundance less than 5, and using the DADA2 module or deblur in QIIME2 software for noise reduction. Compared to existing databases, species annotation can be obtained by selecting the classify sklearn module in QIIME2 software.

The flatness of the box plot can be used to determine whether the sample size is sufficient. As the selected sample size gradually increases, the box plot tends to flatten, indicating that the selected sample size in this study is sufficient, and more sequencing can only discover a small number of new ASVs, which can comprehensively reflect the diversity information of the vast majority of bacteria.

Sample complexity analysis (Alpha Diversity): chao1, dominance, goods_ Coverage,

observed_ Otus, Pielou_ e. Shannon et al. calculated the index using QIIME2 software and plotted the dilution curve and species accumulation box plot.

Beta Diversity: PCoA and NMDS dimensionality reduction plots were plotted using R software, with the Unifrac distance between each sample in the four sample groups calculated using QIIME2 software. Use R software for T-test to determine the species differences among the four sample groups at various taxonomic levels of phyla, class, order, family, genus, and species.

Results

1.Analysis of amplicon sequence variants

Figure S1 presents the cumulative species boxplot for the samples in this study, illustrating how species diversity (i.e., the number of observed ASVs) accumulates with increasing sequencing depth (sample size). A steep upward shift in the boxplot along the horizontal axis indicates rapid discovery of new species—suggesting that current sampling effort is insufficient to capture the full community diversity and that additional sequencing is warranted. Conversely, a flattening or stabilization of the boxplot reflects diminishing returns in species discovery, implying that sampling depth is approaching saturation. As shown in the figure, the boxplot gradually stabilizes as sequencing depth increases, indicating that the selected sample size is sufficient to reliably represent the bacterial community’ s diversity. Further sequencing would yield only a marginal increase in novel ASVs, thereby confirming that the current depth adequately captures the diversity of the majority of bacterial taxa. The next step is to proceed with taxonomic annotation of the identified ASVs.

ASVs taxonomic annotation enables identification of the top 10 microbial genera ranked by

highest relative abundance at the genus level(Figure S2). Based on the species and abundance differences in different samples, the 35 species genera with the highest abundance were selected and clustered at the species level. A heatmap was drawn to reveal the clustering content of species in each sample (Figure S3). There has been a significant change in the abundance of some species after intervention.

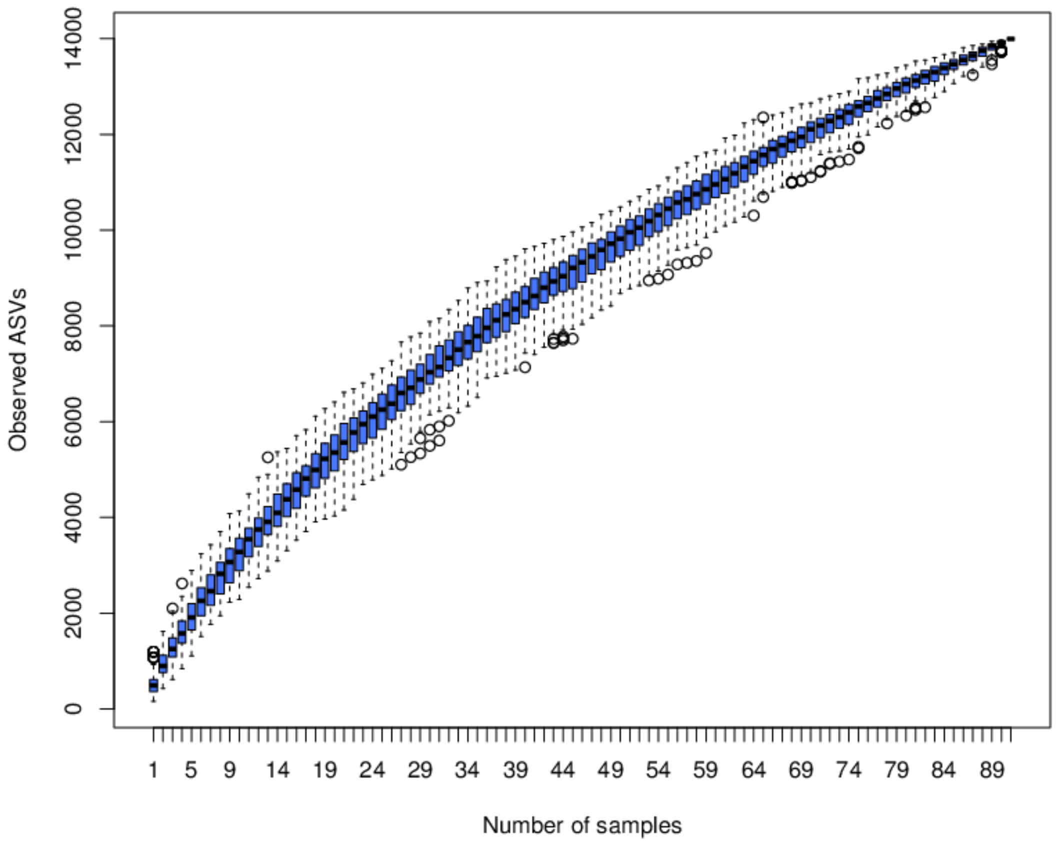

Figure S1. Alpha diversity species accumulation box

Note: Abscissa: number of specimens, ordinate: number of newly discovered ASVs

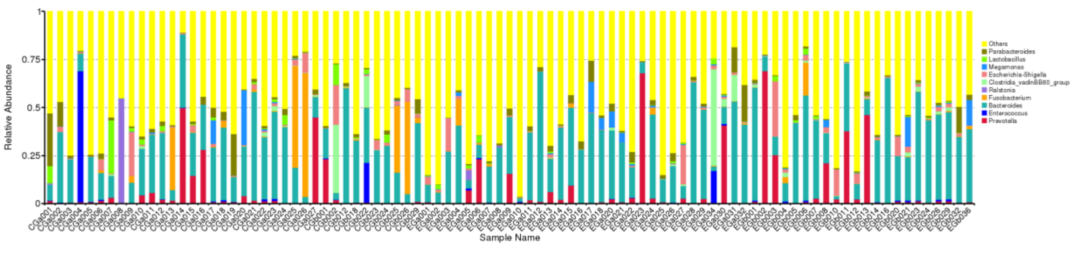

Figure S2. Top10 relative abundations of species

Note: EGa: baseline of exposure group. EGb: Follow-up at the end of week 12 of exposure group. CGa: baseline of control group. CGb: Follow-up at the end of week 12 of control group.Horizontal coordinate: sample name; Ordinate: relative abundance; Different color columns represent different genera of microorganisms. Others represents the sum of relative abundances of all genera except the 10 genera in the figure.

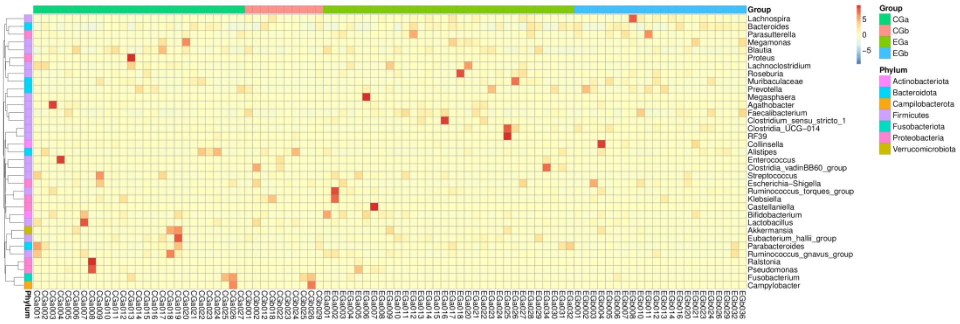

Figure S3. Clustering diagram of relative abundance of species

Horizontal coordinate: sample, vertical coordinate: species annotation information; The value of the heat map is obtained after standardization; The left cluster tree is the species cluster tree.

2.The differences in ASVs between groups

The results from the control group at week 12, along with those from the exposure group, are illustrated in a Venn diagram (Figure S4). This diagram reflects the number of shared amplicon sequence variants (ASVs) between the experimental and control groups after the treatment, as well as the unique ASVs present in each of the three groups at the baseline of the experimental group.A total of 11,699 ASVs were identified across all groups, including 3,911 in the CGb group, 6,635 in the EGa group, and 5,567 in the EGb group. Among the three groups (comprising a total of 906 samples), there were ASVs unique to each group as well as those shared among pairs or all three groups. Specifically, 4,174 ASVs were unique to the EGa group, 2,376 to the EGb group, and 1,640 to the CGb group. Prior to sequencing, all groups underwent identical rarefaction (sparse) processing to normalize sequencing depth, ensuring that the observed differences in microbial

diversity are biologically meaningful. Therefore, this result can suggest that while some bacterial genera were common among the fecal samples of all groups, significant differences in community composition still existed. This variation provides a foundation for identifying and screening specific differential species.

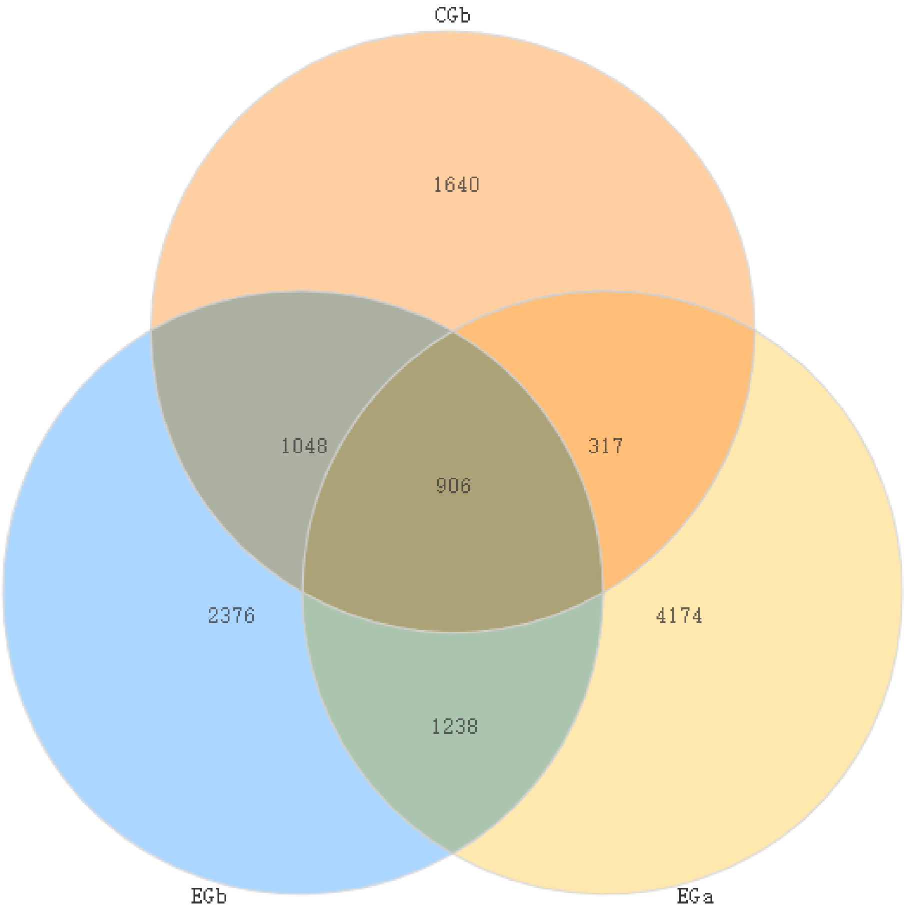

Figure S4. Wayne chart of ASVs differences between groups

3.Differences in dominant species before and after treatment in the expossure group

The top 10 species with the highest average abundance rankings among different taxonomic levels (kingdom, phylum, class, order, family, genus, and species) before and after the exposure in the exposed group, as well as those before exposure in the control group, were selected. The

differences in dominant species were generated into a ternary phase diagram, and the results showed that the differences at the genus level were statistically significant (Figure S5). There will be significant differences in species composition and abundance between groups before and after exposure. Therefore, we can further investigate which bacterial communities have undergone changes.

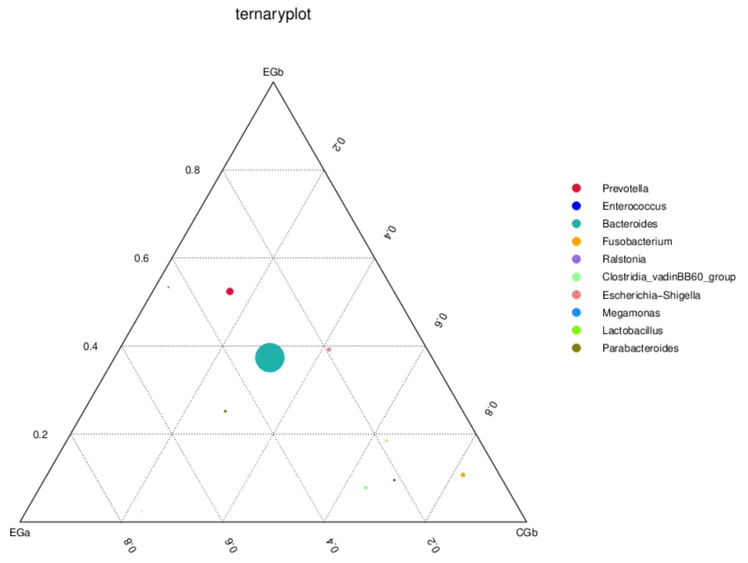

Figure S5. Ternary phase diagram

Note: The three vertices in the figure represent the three sample groups of EGa, EGb, and CGb. The size of the circle is proportional to the abundance, and the color is corresponding to the species. The closer to a vertex, the higher the content of this species in this sample (group).

4.Comparison of demographic data of the subjects who provided stool samples

A comparison of baseline demographic characteristics and clinical symptom assessments revealed no statistically significant differences between the experimental and control groups among participants who provided stool samples.

Variables	Exposure Group (n=33)	Control Group (n=27)	t/χ^2	P value

Age, years	67.09±7.15	70.41±7.62	-1.725	0.09
Gender			0.053	0.958
Male	12 (36.36%)	10 (37.04%)		
Female	21 (63.64%)	17 (62.36%)		
BMI	21.43±2.88	22.45±3.54	-1.209	0.233
Years of education	10.97±3.96	11.89±4.17	-0.803	0.425
AEBQ(FAP)	48.42±3.69	49.30±3.10	-0.995	0.324
AEBQ(FAV)	58.36±3.53	58.70±3.75	-0.359	0.721
MEDAS score	6.70±2.30	6.63±1.45	-1.429	0.162
MMSE score	27.88±4.08	28.04±2.74	-0.179	0.859
GSRS score	36.03±7.00	35.74±4.86	0.189	0.851
SF-LDQ score	11.06±3.83	9.89±3.08	1.313	0.194
SDS score	53.64±8.53	49.63±9.94	1.655	0.104
SAS score	44.88±5.02	42.74±5.98	1.480	0.145

5.Analysis results of demographic factors, mental status, and dietary patterns influencing clinical symptoms

To better control for potential confounding factors, we performed stratified multivariate linear regression analyses, adjusting for covariates known to influence clinical symptoms including age, sex, BMI, years of education, marital status, and dietary habits. The results demonstrated that escitalopram intervention significantly improved both gastrointestinal and emotional symptoms in elderly patients with FGIDs, and this beneficial effect remained robust after accounting for demographic, behavioral, and psychological confounders.

**Variables**	**GSRS**							
	Model 1				Model 2			
	B(SE)	β	t	P	B(SE)	β	t	P
**Intervention**	5.575 (0.502)	0.777	11.095	＜0.001	5.720 (0.551)	0.797	10.373	＜0.001
Gender	-	-	-	-	-0.520(	-0.071	-0.957	0.342

Variables	Model 1 B(SE)	β	t	P	Model 2 B(SE)	β	t	P
					0.543)			
Age, years	-	-	-	-	0.043 (0.033)	0.100	1.335	0.186
BMI	-	-	-	-	-0.012 (0.083)	-0.011	-0.145	0.885
Marriage	-	-	-	-	-0.753 (0.894)	-0.062	-0.843	0.402
Education	-	-	-	-	-0.079 (0.066)	-0.091	-1.207	0.231
AEBQ	-	-	-	-	0.018 (0.046)	0.381	0.371	0.704
MEDAS	-	-	-	-	0.043 (0.138)	0.310	0.310	0.757
Adjusted R^2	0.598				0.591			
F	123.109				14.169			
P	＜0.001				＜0.001			

Variables	SF-LDQ							
	Model 1				Model 2			
	B(SE)	β	t	P	B(SE)	β	t	P
**Intervention**	2.942 (0.348)	0.685	8.452	＜0.001	2.794 (0.383)	0.650	7.289	＜0.001
Gender	-	-	-	-	-0.533 (0.378)	-0.122	-1.412	0.162
Age, years	-	-	-	-	0.012 (0.023)	0.046	0.527	0.600
BMI	-	-	-	-	0.068 (0.058)	0.104	1.173	0.245
Marriage	-	-	-	-	-0.015	0.002	0.024	0.981

					(0.621)			
Education	-	-	-	-	-0.035	-0.068	-0.773	0.442
					(0.046)			
AEBQ	-	-	-	-	0.002	0.058	0.058	0.955
					(0.032)			
MEDAS	-	-	-	-	0.037	0.033	0.383	0.703
					(0.096)			
Adjusted R^2	0.462				0.449			
F	71.432				8.420			
P	＜0.001				＜0.001			

**Variables**	**SDS**							
	Model 1				Model 2			
	B(SE)	β	t	P	B(SE)	β	t	P
**Intervention**	9.365	0.671	8.136	＜0.001	-4.794	0.646	10.373	＜0.001
	(1.151)				(13.911)			
Gender	-	-	-	-	9.022	0.117	1.372	0.174
					(1.238)			
Age, years	-	-	-	-	1.673	0.241	2.781	0.007
					(1.220)			
BMI	-	-	-	-	0.203	0.061	0.690	0.493
					(0.073)			
Marriage	-	-	-	-	1.29	0.044	-0.515	0.608
					(1.87)			
Education	-	-	-	-	-1.032	-0.023	-0.260	0.796
					(2.006)			
AEBQ	-	-	-	-	-0.38	0.067	0.793	0.431
					(1.48)			
MEDAS	-	-	-	-	0.83	0.060	-0.703	0.484

					(1.04)			
Adjusted R^2	0.443				0.456			
F	66.186				8.628			
P	＜0.001				＜0.001			

**Variables**	**SAS**							
	Model 1				Model 2			
	B(SE)	β	t	P	B(SE)	β	t	P
**Intervention**	7.342 (0.727)	0.747	10.104	＜0.001	7.170 (0.794)	0.729	9.030	＜0.001
Gender	-	-	-	-	-0.177 (0.783)	-0.018	-0.227	0.821
Age, years	-	-	-	-	0.075 (0.047)	0.127	1.610	0.1112
BMI	-	-	-	-	0.154 (0.120)	0.103	1.277	0.206
Marriage	-	-	-	-	-1.413 (1.287)	-0.085	-1.098	0.276
Education	-	-	-	-	0.010 (0.095)	0.008	0.107	0.915
AEBQ	-	-	-	-	0.066 (0.067)	0.076	0.989	0.326
MEDAS	-	-	-	-	-0.138 (0.199)	-0.053	-0.693	0.490
Adjusted R^2	0.552				0.548			
F	102.097				12.050			
P	＜0.001				＜0.001			
